# Neuroanatomical and neuropharmacological approaches to postictal antinociception-related prosencephalic neurons: the role of muscarinic and nicotinic cholinergic receptors

**DOI:** 10.1002/brb3.105

**Published:** 2013-04-05

**Authors:** Renato Leonardo de Freitas, Luana Iacovelo Bolognesi, André Twardowschy, Fernando Morgan Aguiar Corrêa, Nicola R Sibson, Norberto Cysne Coimbra

**Affiliations:** 1 Laboratory of Neuroanatomy and Neuropsychobiology, Department of Pharmacology, School of Medicine of Ribeirão Preto, University of São Paulo (USP)Av. dos Bandeirantes 3900, Ribeirão Preto, São Paulo, 14049-900, Brazil; 2 Institute for Neuroscience and Behaviour, Campus Universitarius of Ribeirão Preto of the University of São Paulo (USP)Av. dos Bandeirantes 3900, Ribeirão Preto, São Paulo, 14049-901, Brazil; 3 Laboratory of Central Control of the Arterial Pressure, Department of Pharmacology, School of Medicine of Ribeirão Preto, University of São Paulo (USP)Av. dos Bandeirantes 3900, Ribeirão Preto, São Paulo, 14049-900, Brazil; 4 Experimental Neuroimaging Group, CR-UK/MRC Gray Institute for Radiation Oncology and Biology, Churchill Hospital, University of OxfordHeadington, Oxfordshire OX3 7LJ, Oxford, UK

**Keywords:** cholinergic neurotransmission, dorsal hippocampus, GABAergic neurotransmission, postictal analgesia

## Abstract

Several studies have suggested the involvement of the hippocampus in the elaboration of epilepsy. There is evidence that suggests the hippocampus plays an important role in the affective and motivational components of nociceptive perception. However, the exact nature of this involvement remains unclear. Therefore, the aim of this study was to determine the role of muscarinic and nicotinic cholinergic receptors in the dorsal hippocampus (dH) in the organization of postictal analgesia. In a neuroanatomical study, afferent connections were found from the somatosensory cortex, the medial septal area, the lateral septal area, the diagonal band of Broca, and the dentate gyrus to the dH; all these areas have been suggested to modulate convulsive activity. Outputs to the dH were also identified from the linear raphe nucleus, the median raphe nucleus (MdRN), the dorsal raphe nucleus, and the locus coeruleus. All these structures comprise the endogenous pain modulatory system and may be involved either in postictal pronociception or antinociception that is commonly reported by epileptic patients. dH-pretreatment with cobalt chloride (1.0 mmol/L CoCl_2_/0.2 μL) to transiently inhibit local synapses decreased postictal analgesia 10 min after the end of seizures. Pretreatment of the dH with either atropine or mecamylamine (1.0 μg/0.2 μL) attenuated the postictal antinociception 30 min after seizures, while the higher dose (5.0 μg/0.2 μL) decreased postictal analgesia immediately after the end of seizures. These findings suggest that the dH exerts a critical role in the organization of postictal analgesia and that muscarinic and nicotinic cholinergic receptor-mediated mechanisms in the dH are involved in the elaboration of antinociceptive processes induced by generalized tonic-clonic seizures.

## Introduction

Pentylenetetrazole (PTZ), a gamma amino butyric acid (GABA)ergic receptor antagonist, is commonly used in adult rodents to generate generalized seizure-based animal models of epilepsy (Meilleur et al. 2003; de Freitas et al. 2004; Freitas et al. 2005, 2008, 2009; De Oliveira et al. 2011; Felippotti et al. 2011a,b, 2012). At low doses, PTZ causes absence seizures (Snead 1992), while intermediate doses lead to clonic seizures. High doses of PTZ induce tonic–clonic seizures, eventually leading to generalized status epilepticus (SE) and animal death (Mirski and Ferrendelli 1984). The neuronal substrates for absence seizures have a well-defined thalamus-cortical pathway with no spread to other systems of the brain. Clonic seizures have been shown to arise from the cerebral cortex and forebrain, whereas tonic seizures originate in the brainstem (Browning 1985; Browning and Nelson 1986; Chiu and Burnham 1992; Vergnes and Marescaux 1992, 1994).

Thus, there is evidence that the neurons of the anterior thalamus, retrosplenial cortex, and the dentate gyrus may contribute to the neural network that controls the start of generalized tonic–clonic seizures induced by PTZ (Brevard et al. 2006). Similarly, patterns of PTZ-evoked brain activation, as measured by c-fos expression, encompass the thalamus and hypothalamus at low doses (4 mg/kg) and progressively spread through the brain with increasing dose, culminating in the involvement of the whole brain during tonic–clonic seizures (André et al. 1998; Eells et al. 2004).

The hippocampus has been implicated in the elaboration of epilepsy since the first descriptions of hippocampal formation abnormalities in the 19th century (Goddard et al. 1969). Moreover, following observations that electric stimulation of the dorsal hippocampus (dH) produced an analgesic-like effect (Lico et al. 1974), a role for the hippocampus in antinociception has also been proposed. Furthermore, peripheral painful stimuli activate hippocampal cells, and this response is inhibited by local microinjection of acetylcholine or pilocarpine, both muscarinic cholinergic receptor agonists. In contrast, atropine, a muscarinic cholinergic receptor antagonist, produced the opposite effect (Yang et al. 2007).

Recent findings have demonstrated antinociceptive processes in experimental models of PTZ- or electroshock-induced seizures, in which opioid, 5-HT_2_, muscarinic, and nicotinic cholinergic receptors may be involved (Urca et al. 1981; de Lima and Rae 1991; Coimbra et al. 2001a; De Freitas et al. 2004; Portugal-Santana et al., 2004; Freitas et al. 2005, 2008, 2009).

The hippocampus, rich in cholinergic receptors, is the target of cholinergic fibers from the medial septal area (MSA) and the laterodorsal tegmental nuclei (Lewis et al. 1967; Nicoll, 1985). In addition, cholinergic neurons of the medial septum that project to the hippocampus, the cingulate cortex, and the entorhinal cortex (Metys et al. 1969) receive inputs from a variety of brain and midbrain areas that participate in antinociceptive responses (Basbaum and Fields 1984). Thus, the hippocampus may be an important locus for the modulation of cholinergic neurotransmission involved in postictal analgesia. The aim of this study, therefore, was to determine whether synaptic blockade of the dH, through intrahippocampal microinjection of chloride cobalt, alters postictal analgesia. Subsequently, we elucidated the involvement of muscarinic and nicotinic cholinergic receptors within the dH in the mediation of postictal analgesia induced by seizures.

## Material and Methods

### Animals

Male Wistar albino rats, weighing between 230 and 250 g, from the animal care facility of the University of São Paulo (USP; Campus of Ribeirão Preto) were used. These animals were housed in groups of four in a plexiglass-walled cage, and given free access to food and water throughout the experiment. The room temperature was controlled (22 ± 1°C), and a light–dark cycle (07:00–19:00 h lights on) was maintained. All experiments were performed in accordance with the recommendation of the Committee for Ethics in Animal Experimentation of the FMRP-USP (proc.015/2005) which agrees with the Animal Research Ethics adopted by the Brazilian College of Animal Experimentation (COBEA).

### Nociceptive testing by tail-flick latencies

All rats had their nociceptive thresholds compared using the tail-flick test. Each animal was placed in a restraining apparatus (Stoelting Co., Wood Dale, IL) with acrylic walls, and its tail was placed in a heating sensor (tail-flick Analgesia Instrument; Stoelting, IL), during which time the progressive calorimetric elevation was automatically interrupted the moment the animal removed its tail from the apparatus. The current raised the temperature of the coil (Ni/Cr alloy; 26.04 cm in length × 0.02 cm in diameter) at the rate of 9°C/sec (Prado and Roberts, 1985), starting at room temperature (approximately 20°C). A small current intensity adjustment could be performed, if necessary, at the beginning of the experiment, aiming to obtain three consecutive tail-flick latencies (TFL) between 2.5 and 3.5 sec. If the animal did not remove its tail from the heater within 6 sec, the apparatus was turned off in order to prevent damage to the skin. Three baselines of control TFL were taken at 5-min intervals. TFL were also measured immediately after seizures: 10, 20, 30, 40, 60, 90, 120, 150, and 180 min after the seizures, which were elicited by intraperitoneal (IP) administration of PTZ (64 mg/kg).

### Surgical procedure for neurophysiological and neuropharmacological studies

Animals were anesthetized with sodium pentobarbital (45 mg/kg, IP) and fixed in a stereotaxic frame (David Kopf, Tujunga, CA). A stainless steel guide cannula (outer diameter 0.6 mm, inner diameter 0.4 mm) was implanted in the prosencephalon, targeting the dorsal hippocampus (dH). The upper incisor bar was set at 3.3 mm below the interaural line, such that the skull was horizontal between bregma and lambda. The guide cannula was vertically introduced using the following coordinates, with bregma serving as the reference: anteroposterior, -3.80 mm; mediolateral, 2.5 mm; and dorsoventral, 2.8 mm for the dH. The guide cannula was fixed to the skull using an acrylic resin and two stainless steel screws. At the end of the surgery, each guide cannula was sealed with a stainless steel wire to protect it from obstruction.

### Neuroanatomical study: microinjection of biodextran conjugated to Texas Red in the dH

In four animals previously anesthetized with sodium pentobarbital (45 mg/kg, IP), Texas Red-conjugated biotinylated dextran amine (BDA; biodextran; Molecular Probes, Eugene, OR) at 10% (molecular weight: 3000, volume of 0.2 μL) was unilaterally microinjected into the dH. The upper incisor bar was set at 3.3 mm below the interaural line, such that the skull was horizontal between bregma and lambda. The microneedle (Injex, Ourinhos, São Paulo, Brazil) was vertically introduced into the hippocampus according to the coordinates from Paxinos and Watson's atlas (1997), as mentioned above.

After the injection was completed, a protective layer of sterile gelatin foam was laid in the bone cavity above the pons; the bone was then closed with cyanoacrylate glue thickened with dental cement powder. Seven days after microinjection of the neurotracer, the animal was deeply anesthetized with sodium pentobarbital (45 mg/kg, IP) and transcardially perfused at a rate of 3.5 mL/min with a 20-mL saline followed by 100–200 mL of 2% paraformaldehyde and 2% glutaraldehyde in 0.1 mol/L phosphate buffer, pH 7.4. After fixation, the brains were removed and the mesencephalon was separated from diencephalon, and the pons and medulla oblongata were separated from the spinal cord. The mesencephalon and the prosencephalon were immersed in 10% and 20% sucrose dissolved in 0.1 mol/L phosphate buffer, pH 7.4, at 8°C, for at least 12 h in each solution.

Tissue fragments were immerse in 2-methylbutane (Sigma, London, UK), frozen on dry ice (30 sec), embedded in Tissue Tek O.C.T. compound (Sakura Finetek Europe B.V., the Netherlands), and cut with a cryostat (Leica CM1950, Wetzlar, Germany). All sections were immediately mounted on gelatin-coated slides and stained with hematoxylin–eosin in a robotized autostainer (CV 5030 Leica Autostainer XL, Wetzlar, Germany) to locate the positions of the microinjections sites in a bright-field photomicroscope (AxioImager ZI, Zeiss). The location of the injection site of the Texas Red-labeled biodextran and fluorescent bidirectionally labeled cells were visualized using fluorescence microscopy (AxioImager ZI with APOTOME, Carl-Zeiss-Straße, Oberkochen, Germany).

### Behavioral tests and seizure intensity evaluation

Behavioral tests were performed by placing the rats in the interior of a circular arena, in which the transparent acrylic walls measured 60 cm in diameter and 50 cm in height. The arena was located in an experimental compartment illuminated by a fluorescent lamp (350 lux at the arena floor level). The effects of drug administration (PTZ, atropine, mecamylamine, and physiological saline) were evaluated with the rats inside the arena.

The motor parameters used to evaluate the effect of the Cl^−^ influx and GABA-mediated blockade were the tonic–clonic convulsive reactions induced by IP administration of PTZ at 64 mg/kg. The latency of seizures was defined as the time starting from the injection of PTZ to the first evidence of anterior paw myoclonia, which was considered the first sign of seizures. The severity of the convulsive reactions was evaluated through a modified procedure proposed by De Freitas (2010), which was based on a version of Racine's scores (Racine 1972) that were later modified by Maggio and Gale (1989) (Table 1). The PTZ-induced convulsive reactions were recorded using a video camera (Sony Handycam, New York, NY), and the videos were subsequently evaluated for classification, characterization, and quantification of the convulsive reactions.

**Table 1:** Scale of severity of generalized tonic–clonic convulsive reactions induced by intraperitoneal administration of pentylenetetrazole (64 mg/kg), according to convulsive motor behavior

Score	Seizing reaction
0.0	Exploratory behavior
1.0	Jaw and/or facial myoclonic reaction
	Short duration anterior paw myoclonus
2.0	Head myoclonia
	Moderate myoclonia of anterior paw with duration of at least 5 sec
3.0	Tonic seizures
	Severe anterior paw myoclonic with duration of at least 10 sec
4.0	Tonic seizures
	Rearing and severe myoclonia of the anterior paw
5.0	Tonic seizures
	Rearing and falling, and also myoclonia os anterior and posterior paw

### Neurophysiological study: blockage of synapses in the dH

Five days after surgical implantation of the guide cannula in the dH, a baseline latency of the tail-flick test was obtained in each animal of a given group (*n* = 6–8 per group). Subsequently, each animal received a microinjection of either 0.2 μL of physiological saline (0.9% NaCl) or chloride cobalt (1.0 mmol/0.2 μL) into the dH, or underwent a *sham* procedure that consisted of the introduction of the injector needle into the guide cannula without the microinjection of drugs. After 5 min, the animals received IP administration of PTZ (at 64 mg/kg). TFL were measured immediately and 10, 20, 30, 40, 60, 90, 120, 150, and 180 min after seizures.

### Microinjection of muscarinic and nicotinic cholinergic receptors antagonists

Five days after surgical implantation of the guide cannula in the dH, a baseline latency of the tail-flick test was obtained in each animal. Subsequently, animals were injected in the dH with either physiological saline (0.9% NaCl; 0.2 μL), atropine (1.0 and 5.0 μg/0.2 μL), or mecamylamine (1.0 and 5.0 μg/0.2 μL), followed by IP administration of PTZ (at 64 mg/kg) after 5 min. The nociceptive threshold was measured immediately after and 10, 20, 30, 40, 60, 90, 120, 150, and 180 min after seizures.

### Control of the muscarinic and nicotinic cholinergic antagonists without inducing tonic–clonic seizures

To determine the intrinsic effect of muscarinic and nicotinic cholinergic antagonists on baseline latencies, the tail-flick test was performed in three other groups of animals receiving dH injections of either physiological saline (0.9% NaCl; 0.2 μL) or the higher dose of atropine (5.0 μg/0.2 μL) or mecamylamine (5.0 μg/0.2 μL), followed by IP administration of physiological saline (0.9% NaCl) after 5 min. An evaluation of the effects of drug administration (atropine, mecamylamine, or physiological saline) was performed with the rats inside the arena, recorded over 5 min. The nociceptive threshold was measured 5 min after the rats were placed in an open field, and also 10, 20, 30, 40, 60, 90, 120, 150, and 180 min later.

### Drugs

PTZ (Sigma/Aldrich, St. Louis, MO), cobalt chloride (Sigma), atropine (Sigma), and mecamylamine (Sigma) were each dissolved in physiological saline shortly before use. Ten percent Texas Red-conjugated BDA was also dissolved in physiological saline (BDA: 3000 MW, volume of 0.2 μL; Molecular Probes, Eugene, OR) and was microinjected into the dH.

The diffusion of substances microinjected into the tissue surrounding the injection site in the central nervous system (CNS) is directly proportional to the volume injected (Myers 1966; Routtenberg 1972). According to Myers (1966), volumes of 0.5 μL diffuse an average of 1.04 mm. Injection volumes not higher than 0.2 μL were used in this study to minimize diffusion into the surrounding areas.

### Data analysis

The aim of the above-mentioned experiments was to investigate the effect of seizures on nociceptive thresholds and assess the involvement of dH muscarinic and nicotinic cholinergic neurotransmission in the elaboration of postictal antinociception. To this end, data were collected from experiments and analyzed by analysis of variance (ANOVA) for repeated measurements. To assess significant treatment versus a time interaction, one-way ANOVAs followed by Duncan's post hoc tests were performed at each time interval. A level of *P* < 0.05 was used to confirm statistically significant differences.

## Results

**Figure 1:**
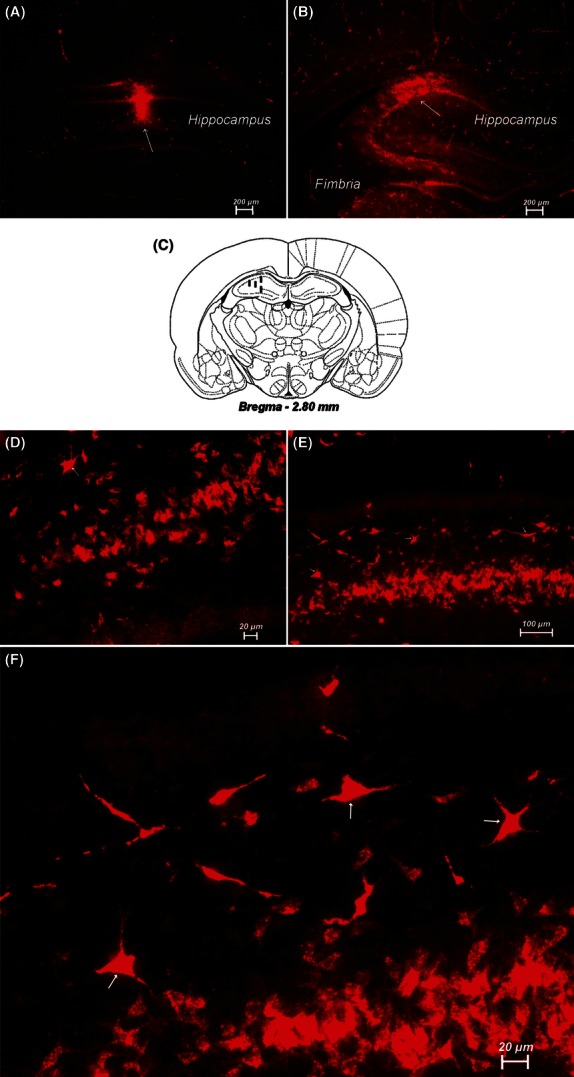
Photomicrographs of coronal sections of the prosencephalon at the level of dorsal hippocampus (dH). (A and B) Sites of microinjections (arrows) of Texas Red-conjugated biodextran. (C) Schematic representation of sites in which the neurotracer microinjections were made into the dH. (D–F) Neurons fluorescently labeled with Texas Red-conjugated biodextran located in the granular layer (D) and in the radial layer (E and F) of the hippocampus contralaterally connected to the dH. Arrows indicate the neural soma.

All microinjections (*n* = 4) of the neurotracer were made into the dH (Fig. 1A–C). Neurotracing showed labeled neurons and fibers situated contralaterally in granular (Fig. 1D) and radial layers (Fig. 1E and F) of the dentate gyrus of the hippocampal formation.

**Figure 2:**
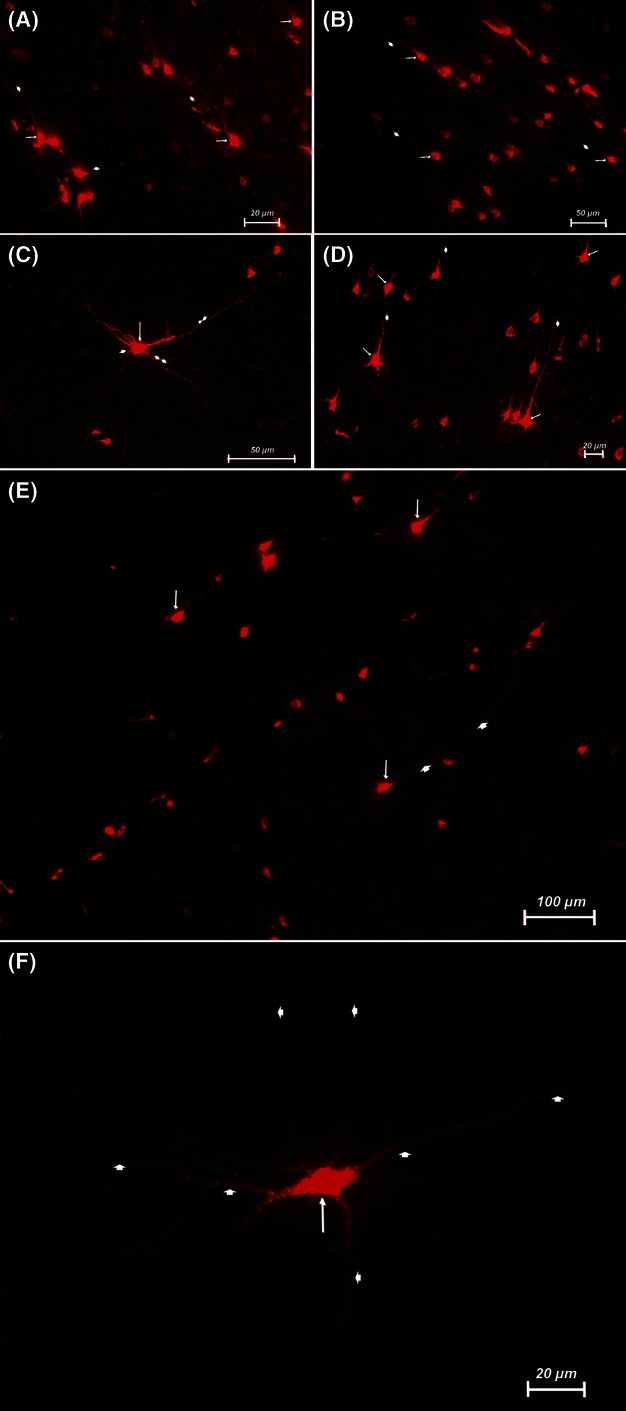
Photomicrographs of coronal sections of the prosencephalon at the level of the primary somatosensory cortex. (A–D) Neurons (arrows) located in the internal pyramidal layer (A and C) and in the external pyramidal layer (B and D) that send axons to the ipsilateral (A and B) and contralateral (C and D) dorsal hippocampus (arrowheads indicate axons and dendrites). (E and F) Neurons (arrows) situated in barrel fields that send axons (arrowheads) to the ipsilateral dorsal hippocampus.

Labeled neurons were also identified in the primary somatosensory cortex, specifically in the pyramidal deep layer (Fig. 2A and C) and in the external pyramidal layer (Fig. 2B and D), ipsilaterally (Fig. 2A and B), or contralaterally (Fig. 2C and D) situated. Labeled neurons were also found contralaterally in the barrel fields (Fig. 2E and F).

**Figure 3:**
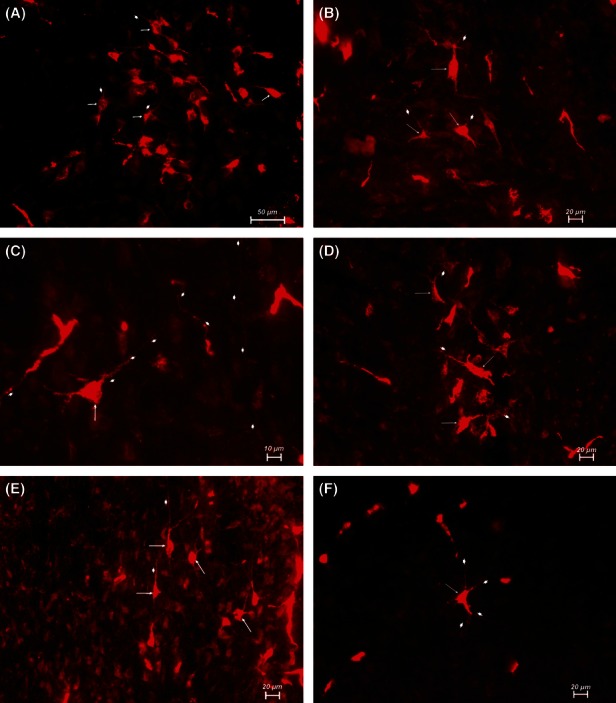
(A–C) Photomicrographs of coronal sections of the prosencephalon, showing neurons fluorescently labeled with Texas Red-conjugated biodextran located in the diagonal band of Broca (A), in the medial septal area (B), and in the lateral septal area (C) that send axons to the ipsilateral dorsal hippocampus. (D–F) Photomicrographs of coronal sections of the brainstem at the level of the raphe nucleus, showing (arrows) neurons labeled with Texas Red-conjugated biodextran in the linear raphe nucleus (D), in the median raphe nucleus (E), and in the dorsal raphe nucleus (F) that send outputs to the dorsal hippocampus. Arrowheads indicate axons and dendrites.

**Figure 4:**
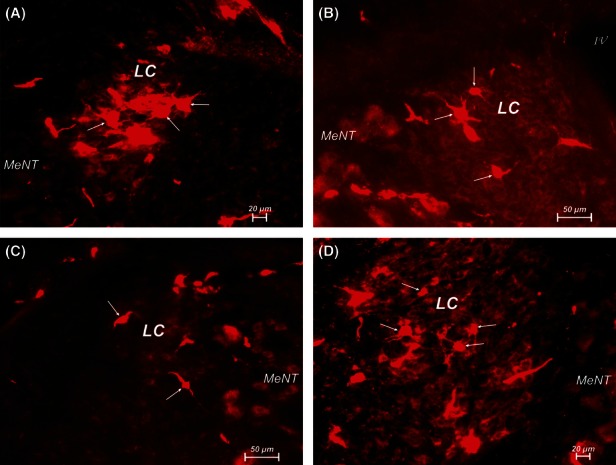
Photomicrographs of coronal sections of the pons at the level of the locus coeruleus (LC) showing labeled neurons (arrows) that send axons ipsilaterally (A and B) and contralaterally (C and D) to the dorsal hippocampus. MeNT: Mesencephalic nucleus of the nervus trigeminus.

Considering more cranial aspects of the forebrain, labeled neurons were ipsilaterally identified in the diagonal band of Broca (DBB) (Fig. 3A), MSA (Fig. 3B), and lateral septal area (LSA) (Fig. 3C). BDA-labeled neurons were also found in the linear raphe nucleus (LRN), MdRN, and dorsal raphe nucleus (DRN) (Fig. 3D, 6E, and 6F, respectively), as well as in the ipsilateral (Fig. 4A and B) and contralateral (Fig. 4C and D) locus coeruleus (LC).

PTZ induced generalized tonic–clonic seizures in all animals. Convulsions after the pretreatment of the dH with the synaptic blocker (cobalt chloride) or the muscarinic or nicotinic cholinergic receptor antagonists (atropine and mecamylamine, respectively) were not preceded by wild running. The duration of tonic–clonic seizures varied from 10 to 13 sec in the saline plus PTZ-treated group, from 9 to 16 sec in the sham procedure plus PTZ-treated group, and from 9 to 12 sec in the cobalt chloride plus PTZ-treated group, as shown in Table 2. The duration of seizures ranged from 9 to 23 sec in the atropine plus PTZ-treated groups (Table 3, A), and ranged from 8 to 18 sec in the mecamylamine plus PTZ-treated groups (Table 3, B), and ranged from 9 to 20 sec in the control groups (Table 3, A and B).

**Table 2:** Lack of effect after synaptic inactivation of the dorsal hippocampus (dH) inputs with chloride cobalt on the latency of first myoclonia, duration of each tonic–clonic seizure, total duration of seizures, the severity of tonic–clonic convulsions, and on the temporal window covering the time between the first and the last seizure induced by intraperitoneal (IP) administration of pentylenetetrazole (PTZ) (64 mg/kg)

		Duration (mean ± SEM) of each tonic–clonic seizure (sec)						
					
Group (*N* = 6–8)	Latency (mean ± SEM) of starting the first myoclonia (sec)	1st	2nd	3rd	4th	Total duration (mean ± SEM) of seizures (sec)	Severity (mean ± SEM) of tonic–clonic seizures (scale from 0 to 5)	Temporal window (mean ± SEM) covering the time between the first and the last seizure
Sham intrahippocampal + PTZ (64 mg/kg, IP)	60 ± 5	11 ± 3	11 ± 2	16 ± 3	9 ± 2	47 ± 3	3.7 ± 0.2	5 min and 3 ± 12 sec
Saline (NaCl 0.9%/0.2 μL) intrahippocampal + PTZ (64 mg/kg, IP)	52 ± 8	10 ± 2	13 ± 2	10 ± 2		33 ± 5	3.8 ± 0.4	5 min and 50 ± 15 sec
Cobalt chloride (1.0 mmol/0.2 μL) intrahippocampal + PTZ (64 mg/kg, IP)	58 ± 6	12 ± 2	9 ± 1	11 ± 2	11 ± 2	41 ± 2	3.8 ± 0.3	5 min and 40 ± 22 sec

**Table 3:** Lack of effect of dorsal hippocampus (dH) pharmacological treatments with atropine (A) and mecamylamine (B) at different doses on the latency of first myoclonia, duration of each tonic–clonic seizure, total duration of seizures, the severity of tonic–clonic convulsions, or on the temporal window covering the time between the first and the last seizure induced by intraperitoneal (IP) administration of pentylenetetrazole (PTZ) (64 mg/kg)

		Duration (mean ± SEM) of each tonic–clonic seizure (sec)						
					
	Latency (mean ± SEM) of starting the first myoclonia (sec)	1st	2nd	3rd	4th	Total duration (mean ± SEM) of seizures (sec)	Severity (mean ± SEM) of tonic–clonic seizures (scale from 0 to 5)	Temporal window (mean ± SEM) covering the time between the first and the last seizure
Group A (*N* = 6–8)								
Saline (NaCl 0.9%/0.2 μL) intrahippocampal + PTZ (64 mg/kg, IP)	44 ± 7	10 ± 3	20 ± 3	14 ± 3	10 ± 4	54 ± 3	3.3 ± 0.2	5 min and 23 ± 21 sec
Atropine (1.0 μg/0.2 μL) intrahippocampal + PTZ (64 mg/kg, IP)	58 ± 8	11 ± 3	23 ± 4	9 ± 4	10 ± 3	53 ± 4	3.7 ± 0.3	5 min and 35 ± 16 sec
Atropine (5.0 μg/0.2 μL) intrahippocampal + PTZ (64 mg/kg, IP)	53 ± 7	13 ± 2	18 ± 3	15 ± 3	9 ± 2	55 ± 3	3.4 ± 0.2	5 min and 17 ± 15 sec
Group B (*N* = 7–9)								
Saline (NaCl 0.9%/0.2 μL) intrahippocampal + PTZ (64 mg/kg, IP)	49 ± 5	13 ± 2	18 ± 4	12 ± 4	9 ± 3	52 ± 3	3.5 ± 0.2	5 min and 23 ± 10 sec
Mecamylamine (1.0 μg/0.2 μL) intrahippocampal + PTZ (64 mg/kg, IP)	62 ± 6	11 ± 2	21 ± 3	15 ± 3	8 ± 3	55 ± 3	3.3 ± 0.3	4 min and 51 ± 10 sec
Mecamylamine (5.0 μg/0.2 μL) intrahippocampal + PTZ (64 mg/kg, IP)	53 ± 6	18 ± 3	15 ± 3	10 ± 2	9 ± 4	52 ± 3	3.6 ± 0.3	5 min and 18 ± 20 sec

The severity of seizures induced by IP administration of PTZ (at 64 mg/kg) was assessed according to a severity index based on the motor impairment induced by tonic and/or clonic responses (shown in Table 1). The seizure intensity was not altered by different treatments and the scores ranged from 3.7 to 3.8 in the chloride cobalt pretreated group (Table 2), from 3.3 to 3.7 in the atropine pretreated group (Table 3, A), and from 3.3 to 3.6 in the mecamylamine pretreated group (Table 3, B).

Blocking synapses in the dH decreased the antinociception that followed tonic–clonic seizures. The treatment showed significant effects (*F* (3,38) = 23.93; *P* < 0.01), time (*F* (9,20) = 544.67; *P* < 0.001), and the treatment versus time interaction (*F* (27,56) = 20.17; *P* < 0.001) were also significant. Repeated-measure ANOVAs showed a significant treatment effect of postictal analgesia from 0 to 90 min after seizures (*F* (3,28), varying from 6.69 to 16.66; *P* < 0.01).

**Figure 5:**
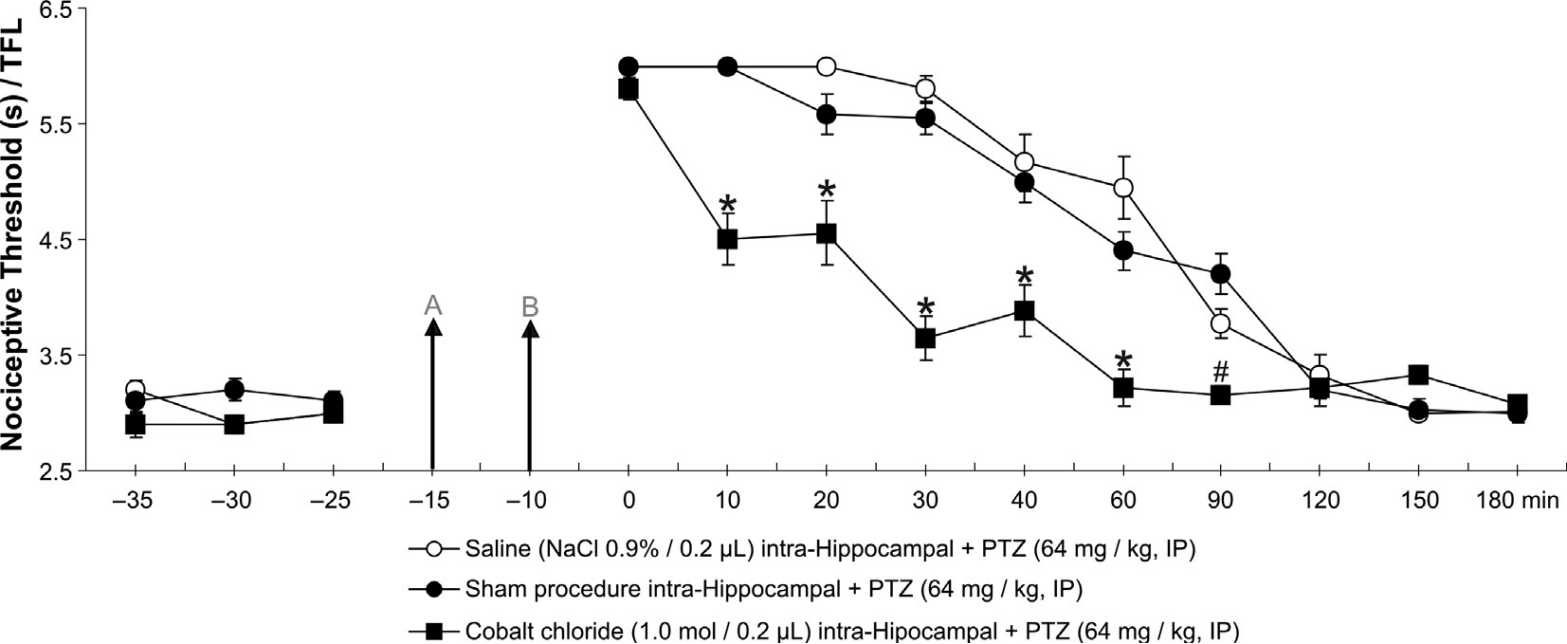
Effect of microinjection into the dorsal hippocampus of (▪–▪) chloride cobalt (1.0 mmol/L per 0.2 μL), followed 5 min after by IP injection of pentylenetetrazole (PTZ; 64 mg/kg, IP) on postictal analgesia, *n* = 6–8; *P < 0.05; according to MANOVA, followed by a Duncan post hoc test, compared with the control ([•–•] intrahippocampal sham procedure followed by IP PTZ). #Statistically significant differences compared with (○–○) intrahippocampal-saline-treated group followed by IP PTZ. Increases in nociceptive threshold were presented as means ± SEM. Arrow points in (A) represent the microinjection sites of either physiological saline, chloride cobalt, or sham procedure into the dH; arrow points in (B) represent IP administration of PTZ 5 min after each above-mentioned microinjection in the dorsal hippocampus. TFL, tail-flick latencies.

Post hoc analyses showed that blocking synapses in the dH with cobalt chloride at 1.0 mmol/0.2 μL decreased the postictal antinociception when compared with the sham procedure group or to the saline-treated group followed by IP PTZ administration (at 64 mg/kg) (data shown in Fig. 5).

**Figure 6:**
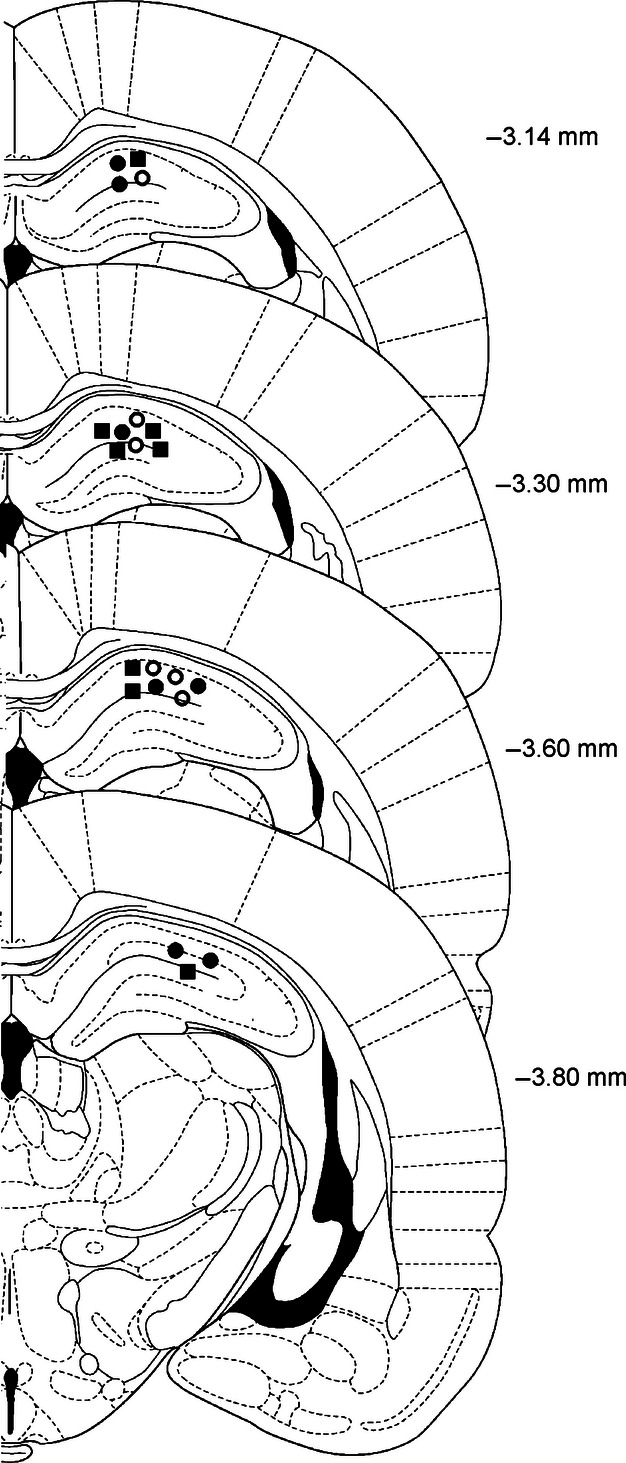
Schematic representation of histologically confirmed sites of microinjections in the dorsal hippocampus of (▪) chloride cobalt, (○) saline, and (•) sham procedure followed by IP PTZ treatment in anagrams of Paxinos and Watson's atlas (1997).

Microinjection sites for the neurophysiological study were all verified to be located in the dH, as shown in Fig. 6. To evaluate the involvement of the muscarinic cholinergic system of the hippocampal formation in the organization of postictal analgesia, atropine was microinjected into the dH. There were significant effects of this treatment (*F* (2,19) = 65.11; *P* < 0.001), time (*F* (9,11) = 64.11; *P* < 0.001), and the treatment versus time interaction (*F* (18,20) = 10.53; *P* < 0.001). One-way ANOVAs showed a significant treatment effect of postictal analgesia from 0 to 90 min after seizures (*F* (2,19), varying from 2.82 to 81.30; *P* < 0.05).

**Figure 7:**
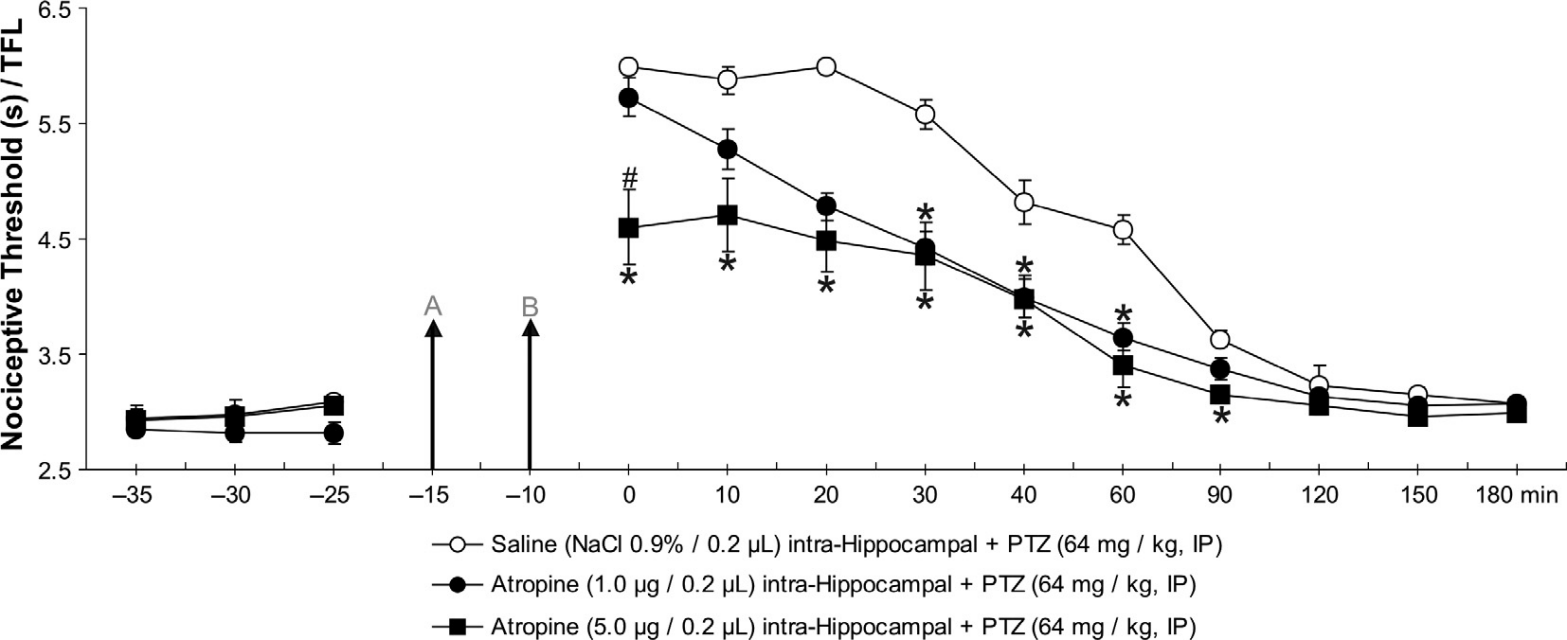
Effect of microinjection into dorsal hippocampus of (•–•) atropine at 1.0 μg/0.2 μL or (▪–▪) atropine at 5.0 μg/0.2 μL, followed, after 5 min, by intraperitoneal (IP) administration of pentylenetetrazole (PTZ; 64 mg/kg, IP) on postictal analgesia; *n* = 6–8; *P < 0.05; according to MANOVA, followed by the Duncan post hoc test, compared with the control ([○–○] intrahippocampal-saline-treated group followed by IP PTZ). #Statistically significant differences, compared with atropine injection at 1.0 μg/0.2 μL intrahippocampal + IP PTZ. Increases in nociceptive thresholds were presented as means ± SEM. Arrow points in (A) represent the microinjection of either physiological saline or atropine and arrow points in (B) represent the IP administration of PTZ 5 min after each above-mentioned microinjection in the dorsal hippocampus. TFL, tail-flick latencies.

Post hoc analyses showed that the intrahippocampal administration of atropine at the lower concentration (1.0 μg/0.2 μL) decreased the postictal analgesia 20 min after the end of seizures. However, the microinjection of atropine at the highest concentration (5.0 μg/0.2 μL) decreased the postictal analgesia immediately after the end of seizures (Fig. 7).

To evaluate the action of intrahippocampal nicotinic cholinergic receptor antagonism on postictal antinociception, the dH was pretreated with mecamylamine at 1.0 and 5.0 μg/0.2 μL. There were significant effects of this treatment (*F* (2,20) = 10.12; *P* < 0.01), time (*F* (9,12) = 54.62; *P* < 0.001), and the treatment versus time interaction (*F* (18,22) = 1.43; *P* < 0.05). One-way ANOVAs showed that there were significant treatment effects from 0 to 120 min (*F* (2,20) varying from 0.50 to 9.32; *P* < 0.01).

**Figure 8:**
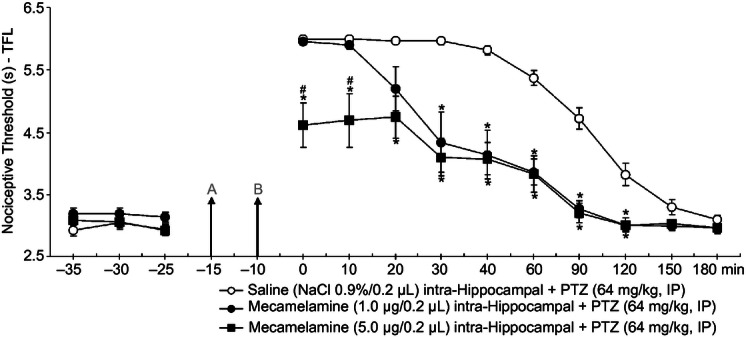
Effect of microinjection into dorsal hippocampus of (•–•) mecamylamine at 1.0 μg/0.2 μL or (▪–▪) mecamylamine at 5.0 μg/0.2 μL, followed, after 5 min, by intraperitoneal (IP) administration of pentylenetetrazole (PTZ; 64 mg/kg, IP) on postictal analgesia; *n* = 6–8; *P < 0.05; according to MANOVA, followed by the Duncan post hoc test, compared with the control ([○–○] intrahippocampal-saline-treated group followed by IP PTZ). #Statistically significant differences compared with mecamylamine injection at 1.0 μg/0.2 μL) intrahippocampal + IP PTZ. Increases in nociceptive threshold were presented as means ± SEM. Arrow points in (A) represent the microinjection site of either physiological saline or mecamylamine, and in (B), arrow points represent the IP administration of PTZ 5 min after each above-mentioned microinjection into the dorsal hippocampus. TFL, tail-flick latencies.

Post hoc analyses showed that intrahippocampal administration of mecamylamine at the lower concentration (1.0 μg/0.2 μL) decreased the postictal analgesia 30 min after the end of seizures. However, microinjection of atropine at the highest concentration (5.0 μg/0.2 μL) decreased the postictal analgesia immediately after the end of the seizures (Fig. 8).

**Figure 9:**
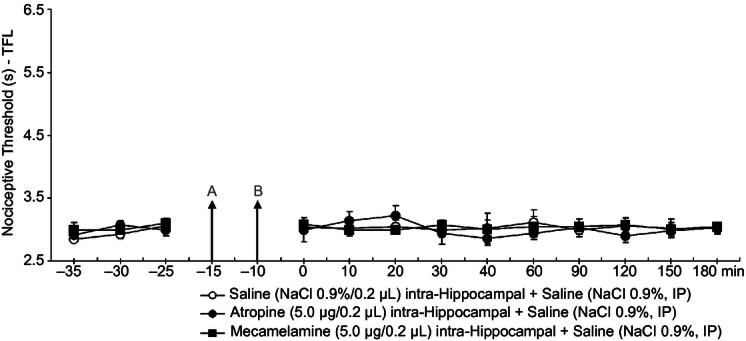
Lack of effect of microinjection into the dorsal hippocampus of (○–○) physiological saline, (•–•) atropine at 5.0 μg/0.2 μL, or (▪–▪) mecamylamine at 5.0 μg/0.2 μL, followed, after 5 min, by IP administration of saline (0.9% NaCl; IP) on nociceptive threshold; *n* = 7–9; nociceptive thresholds were presented as means ± SEM. Arrow points in (A) represent the IP-administration site of either physiological saline, atropine, or mecamylamine and in (B), arrow points represent the IP administration of physiological saline 5 min after each above-mentioned microinjection in the dorsal hippocampus. TFL, tail-flick latencies.

To determine the effect of atropine and mecamylamine at the highest concentration (5.0 μg/0.2 μL) on baseline TFL, central microinjections of each pharmacological antagonist was followed by peripheral administration of physiological saline. No statistically significant effects on nociceptive thresholds were found (Fig. 9).

**Figure 10:**
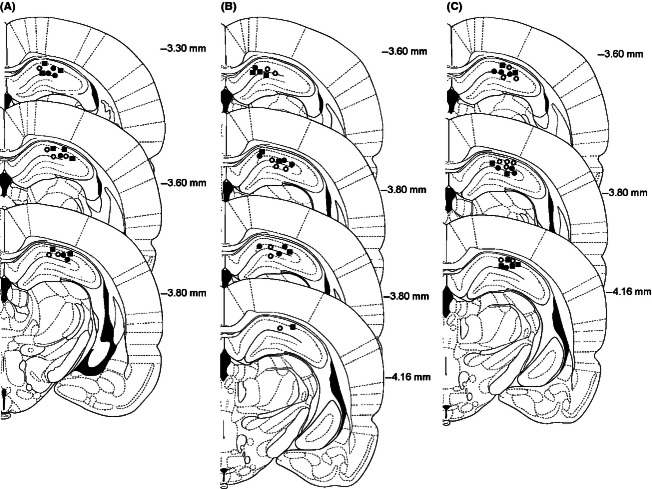
Schematic representation of histologically confirmed sites of microinjections in the dorsal hippocampus of (○) saline, (•) atropine at 1.0 μg/0.2 μL, or (▪) atropine at 5.0 μg/0.2 μL followed by intraperitoneal (IP) administration of pentylenetetrazole (PTZ) (A); or (○) saline, (•) mecamylamine at 1.0 μg/0.2 μL, or (▪) mecamylamine at 5.0 μg/0.2 μL followed by IP PTZ (B); (○) saline, (•) atropine at 5.0 μg/0.2 μL, or (▪) mecamylamine at 5.0 μg/0.2 μL followed by IP saline (C), in anagrams of Paxinos and Watson's atlas (1997).

Microinjection sites for the neuropharmacological study were all verified to be located in the dH, as shown in Fig. 10.

## Discussion

Considering that the blockade of GABA-meditated chloride influx with PTZ caused tonic and tonic–clonic convulsions in all animals in this investigation, GABAergic inputs seem to exert a tonic inhibition of neurons involved in motor responses. Seizures induced by GABAergic dysfunction were followed by persistent postictal antinociception and the present findings show evidence that the dH exerts a putative role in this phenomenon.

Using a neuroanatomical approach, we have demonstrated connections between the dH and areas thought to be involved in the elaboration of epileptogenic activity, such as the primary somatosensory cortex, the barrel field somatosensory cortex, the DBB, the MSA, the LSA, and the regions of granular and radial layers of the dentate gyrus of the hippocampal formation. We also identified connections between the dH and the LRN, the MdRN, the DRN, and the LC, all of which are important areas for the activation of antinociceptive processes following tonic–clonic seizures. Recent evidence suggests that the hippocampus may have important roles in the central perception of pain and the CA3 field of the hippocampal formation has been suggested to be involved in nociceptive perception (Ma et al. 2012).

Although part of the present neuroanatomical findings can be considered confirmatory data (Köhler and Steinbusch 1982; Schober and Fischer 1991; Acsády et al. 1996), the study of these connections in the context of the organization of postictal antinociception and seizures is very important, considering that seizure-induced impairments in sensory cortex function may contribute to ictal and interictal behavioral anomalies during epilepsy (Petersen 2007; Vuong et al. 2011).

A number of neurotransmitters and neuromodulators have been implicated in the hippocampal pathways involved in pain and analgesia, such as those of the muscarinic cholinergic system (Jiao et al. 2009). At the same time, glutamatergic and serotonergic agents, when administered through intrahippocampal and intradentate gyrus routes, induce antinociception in rats (McKenna & Melzack, 2001; Soleimannejad et al., 2006, 2007). In addition, the cholinergic neurotransmission and the muscarinic receptors of the hippocampus can be involved in the modulation of the nociceptive response by regulating the electrical activities of pain-excited or -inhibited neurons in the hippocampal CA3 field of normal rats submitted to electrical stimulation of the ischial nerve, a model of neuropathic pain (Li et al. 2011).

We have previously shown that the DRN and its serotonergic receptors, as well as the LC and its noradrenergic receptors, are key structures in the pain endogenous modulatory system recruited during postictal antinociception (Ferreira et al. 2005; Freitas et al. 2005, 2008, 2009; Felippotti et al. 2011a). Thus, the outputs to the dH from the endogenous pain modulatory system nuclei shown in this study may reflect their involvement in postictal antinociception through the modulation of the activity of hippocampal outputs.

The varicose monoaminergic, glutamatergic, and cholinergic axons are equipped with neuronal nicotinic acetylcholine receptors and these nonsynaptically localized receptors are of high affinity. The activation of the receptor itself causes depolarization of the neuron, release of chemical transmitters, and may result in the modulation of neurotransmitter release initiated by axonal firing (Vizi and Lendvai 1999). The modulation of presynaptic nicotinic receptors in the neurochemical circuitry of the brain and brainstem areas that are interconnected with the dH, as presently shown, can provide a cholinergic mechanism that recruits other nuclei of the brainstem involved in monoaminergic descending control of pain and can activate complex neuronal assemblies that elaborate epileptogenic activity in the CNS, resulting in subsequent postictal antinociception (Coimbra et al. 2001a,b; Shouse et al. 2001; De Freitas et al. 2004; Ferreira et al. 2005; Freitas et al. 2005, 2008, 2009; De Oliveira et al. 2006, 2011; Felippotti et al. 2011a,b, 2012).

The present data corroborate findings from Klamt and Prado (1991), who showed that the microinjection of carbachol, a muscarinic cholinergic receptor agonist, into the hippocampus causes antinociception and decreases pain-related behavior in rats (Klamt and Prado 1991). In fact, in the current study, PTZ caused tonic–clonic seizures followed by antinociception, which was decreased by the inactivation of the dH and by the local blockade of cholinergic muscarinic and nicotinic receptors in the dH. These findings support the concept that the neurotransmitter acetylcholine may be involved in the antinociceptive process in seizures that are evoked by GABA-mediated Cl^−^ influx blockade. Given that the administration of cholinergic muscarinic and nicotinic receptors antagonists at the highest dose did not exert a significant influence on the baseline nociceptive threshold of the rodents, our data indicate that the intrahippocampal effects of these drugs were due to their specific action on postictal antinociception.

A recent report showed that pretreatment with bicuculline partially prevents nicotine-induced antinociception, suggesting that the GABA_A_ receptor may contribute to acetylcholine-mediated analgesia (Mui et al. 1997). Others have suggested that the GABAergic system may modulate nicotinic receptor-mediated seizures (Dobelis et al. 2003).

Interestingly, in this study, transitory synaptic blockade of the dH with cobalt chloride, which specifically blocks neuronal Ca^++^ influx (Kretz 1984), as well as intrahippocampal pretreatment with cholinergic muscarinic and nicotinic receptor antagonists did not alter seizure severity. We tried to avoid the spreading of drugs to other regions of the hippocampus by restricting the volume of microinjections to 0.2 μL. Considering this, our findings suggest that the severity of convulsive reactions induced by peripheral treatment with PTZ, rather than cholinergic mechanisms of the dH, may also be mediated by other areas of the CNS, for example, the entorhinal cortex (Stringer 1996), amygdaloid complex (Galvis-Alonso et al. 2004; Foresti et al. 2008), other structures of the hippocampal formation (Rodrigues et al. 2004; Lan et al. 2007), and the corpora quadrigemina (Garcia-Cairasco 2002; Doretto et al. 2009). Additionally, the severity of convulsive reactions may possibly be modulated by the substantia nigra, pars reticulata, and inhibitory pathways that modulate aversive stimulus-induced defensive responses (Coimbra and Brandão 1993; Eichenberger et al. 2002; Ribeiro et al. 2005; Castellan-Baldan et al. 2006), as well as epileptogenic activity (Rodrigues et al. 2004; Rossetti et al. 2011, 2012).

In conclusion, the neuroanatomical substrates identified in the present work reinforce the involvement of the pain inhibitory system in pain control and suggest that the nuclei connected to the dH are critically involved in the elaboration of postictal antinociception. A reduction in dH activity, caused by blocking local synapses with cobalt chloride, decreased postictal analgesia, confirming dH involvement in the elaboration of antinociception induced by tonic-clonic seizures. Furthermore, our findings suggest that the muscarinic and nicotinic cholinergic pathways from the dH exert an important role in the organization of postictal antinociception, possibly modulated by input to the dH from both prosencephalic areas and from the endogenous pain inhibitory system. Elucidation of the neurochemistry of the antinociceptive process evoked by convulsive seizures may represent an important step toward understanding the neural basis of the control of pain.
